# Low-Level Arsenic Impairs Glucose-Stimulated Insulin Secretion in Pancreatic Beta Cells: Involvement of Cellular Adaptive Response to Oxidative Stress

**DOI:** 10.1289/ehp.0901608

**Published:** 2010-01-25

**Authors:** Jingqi Fu, Courtney G. Woods, Einav Yehuda-Shnaidman, Qiang Zhang, Victoria Wong, Sheila Collins, Guifan Sun, Melvin E. Andersen, Jingbo Pi

**Affiliations:** 1 Division of Translational Biology, The Hamner Institutes for Health Sciences, Research Triangle Park, North Carolina, USA; 2 School of Public Health, China Medical University, Shenyang, China; 3 Division of Computational Biology and; 4 Flow Cytometry and Confocal Core, The Hamner Institutes for Health Sciences, Research Triangle Park, North Carolina, USA

**Keywords:** arsenic, diabetes, insulin secretion, Nrf2, oxidative stress, pancreatic beta cells, ROS

## Abstract

**Background:**

Chronic exposure of humans to inorganic arsenic, a potent environmental oxidative stressor, is associated with incidence of type 2 diabetes (T2D). A key driver in the pathogenesis of T2D is impairment of pancreatic β-cell function, with the hallmark of β-cell function being glucose-stimulated insulin secretion (GSIS). Reactive oxygen species (ROS) derived from glucose metabolism serve as one of the metabolic signals for GSIS. Nuclear factor-erythroid 2–related factor 2 (Nrf2) is a central transcription factor regulating cellular adaptive response to oxidative stress.

**Objectives:**

We tested the hypothesis that activation of Nrf2 and induction of antioxidant enzymes in response to arsenic exposure impedes glucose-triggered ROS signaling and thus GSIS.

**Methods and results:**

Exposure of INS-1(832/13) cells to low levels of arsenite led to decreased GSIS in a dose- and time-dependent fashion. Consistent with our hypothesis, a significantly enhanced Nrf2 activity, determined by its nuclear accumulation and induction of its target genes, was observed in arsenite-exposed cells. In keeping with the activation of Nrf2-mediated antioxidant response, intracellular glutathione and intracellular hydrogen peroxide–scavenging activity was dose dependently increased by arsenite exposure. Although the basal cellular peroxide level was significantly enhanced, the net percentage increase in glucose-stimulated intracellular peroxide production was markedly inhibited in arsenite-exposed cells. In contrast, insulin synthesis and the consensus GSIS pathway, including glucose transport and metabolism, were not significantly reduced by arsenite exposure.

**Conclusions:**

Our studies suggest that low levels of arsenic provoke a cellular adaptive oxidative stress response that increases antioxidant levels, dampens ROS signaling involved in GSIS, and thus disturbs β-cell function.

Type 2 diabetes (T2D) has become a serious public health problem throughout the world. Zimmet et al. (2001) estimated that approximately 150 million people worldwide had T2D in the year 2000, and they predicted that this number could double by 2025. In the United States alone, T2D affected > 7% of the population and resulted in an estimated $174 billion total annual economic cost in 2007 (American Diabetes Association 2008). Obesity has been recognized as the leading cause of T2D, whereas many factors, including genetic elements and lifestyle, are involved in the incidence of obesity. However, a link between environmental exposures and diabetes has also been established (Navas-Acien et al. 2008) but has received little attention by the medical community.

Arsenic is a naturally occurring element that is ubiquitous in the environment in both organic and inorganic forms (Nordstrom 2002). Human exposure to the generally more toxic inorganic arsenic (iAs) occurs in occupational or environmental settings, as well as through medicinal arsenical use (Abernathy et al. 1999). The main source of human environmental exposure is through consumption of water containing elevated levels of arsenic, primarily from natural contamination (Gebel 1999). The U.S. Environmental Protection Agency set the arsenic standard for drinking water at 0.01 ppm in 2006. An estimated 13 million Americans were exposed to higher levels of arsenic (> 0.01 ppm) through public water systems by 2006.

Chronic exposure to high levels of iAs is associated with a wide range of human ailments including cancer, arteriosclerosis, hypertension, and T2D. Although the evidence for a causal association between iAs exposure and T2D is not unequivocally established (Navas-Acien et al. 2006), epidemiological studies carried out in Taiwan (Chiu et al. 2006), Bangladesh (Nabi et al. 2005), Sweden (Rahman et al. 1996), and Mexico (Coronado-Gonzalez et al. 2007) have shown a strong diabetogenic effect of arsenic in humans. More recently, Meliker et al. (2007) reported a modest but significant association between iAs exposure and T2D in residents of Michigan, with average iAs level in drinking water of 0.011 ppm. In addition, a cross-sectional study carried out in 788 adults who participated in the 2003–2004 National Health and Nutrition Examination Survey reveals a strong positive association between low-level arsenic exposure and the prevalence of T2D in the United States (Navas-Acien et al. 2008). These new epidemiological studies provided additional support for the importance of arsenic exposure in the development of T2D. Although the precise mechanisms for the diabetogenic effect of arsenic are still largely undefined, recent *in vitro* and *in vivo* experimental studies indicated that iAs or its metabolites impair insulin-dependent glucose uptake and result in insulin resistance (Izquierdo-Vega et al. 2006; Paul et al. 2007). In isolated rat islets, iAs decreased insulin transcription and secretion (Diaz-Villasenor et al. 2006).

A key driver in the pathogenesis of T2D is the impairment of pancreatic β-cell function, with the hallmark of β-cell function being glucose-stimulated insulin secretion (GSIS) (Fridlyand and Philipson 2004). According to the currently accepted hypothesis, the control of GSIS in β cells depends largely on glucose metabolism, in which glycolytic and oxidative phosphorylation triggers a sequence of signaling events, including increased ATP production and ATP/ADP ratio, leading to insulin secretion (Henquin 2004; Newgard and McGarry 1995). Emerging evidence—including our own (Pi et al. 2007)—suggests that, in addition to ATP and ATP/ADP ratio, reactive oxygen species (ROS), such as hydrogen peroxide (H_2_O_2_), derived from glucose metabolism serve as one of the metabolic signals for GSIS. Thus, endogenous antioxidant enzymes that can be robustly induced in response to oxidative stress have the potential to blunt such a glucose-triggered ROS signal and inhibit GSIS (Pi et al. 2010; Zhang et al. 2010).

Accumulating data, including our previous studies (Pi et al. 2003a, 2003b), suggest that arsenic exposure is associated with increased oxidative stress, which has been undisputedly implicated in the etiology of T2D. A key cellular component that defends cells against oxidative damage is nuclear factor-erythroid 2–related factor 2 (Nrf2), a transcription factor that regulates both constitutive and inducible expression of many antioxidant/detoxification enzymes (Kobayashi and Yamamoto 2006). However, this same Nrf2-driven induction of endogenous antioxidant enzymes, meant to maintain intracellular redox homeostasis and limit oxidative damage, may also have the potential, as a side effect, to diminish ROS that function as intracellular signals (Pi et al. 2010; Zhang et al. 2010). Here we report that low levels of arsenite trigger a cellular adaptive oxidative stress response and impair ROS signaling involved in GSIS, thus disturbing β-cell function.

## Materials and Methods

### Cell culture and reagents

INS-1(832/13) cells were kindly provided by C. Newgard (Duke University, Durham, NC, USA) and were cultured in RPMI medium 1640 supplemented with 10% fetal bovine serum (FBS), 10mM glucose, 25 mM HEPES, 2 mM l-glutamine, 50 μM β-mercaptoethanol, 100 U penicillin/mL, and 100 μg streptomycin/mL. Cultures were maintained at 37°C in a humidified 5% CO_2_ atmosphere. We used passages 55–59 at 75–90% confluence for the present study; there was no difference in the glucose responsiveness of the cells among passages 55, 56, 57, 58, and 59. Culture media, FBS, and supplements were purchased from Invitrogen (Carlsbad, CA, USA). Sodium arsenite, fatty acid–free bovine serum albumin (BSA), β-mercaptoethanol, and glucose solution (45%) were obtained from Sigma (St. Louis, MO, USA).

### Measurement of insulin secretion

Experiments were performed in static incubation as described previously (Pi et al. 2007). Levels of secreted insulin were normalized to DNA content, which was determined by an overnight incubation at 37°C with a lysis buffer containing 30 mM Tris-HCl, 10 mM EDTA, 1% sodium dodecyl sulfate, and 50 μg/mL proteinase K (Qiagen, Valencia, CA, USA), followed by a measurement of absorbance at 260 nm using a Nanodrop spectrophotometer (Thermo Scientific, Inc., Wilmington, DE, USA). Insulin measurements were determined using a radioimmunoassay kit (Linco Research, St. Charles, MO, USA) with rat insulin as the standard.

### Antioxidant response element (ARE) reporter assay

Cignal Lenti ARE reporter lentiviral particles were obtained from SABiosciences (Frederick, MD, USA). Lentiviral transduction of INS-1(832/13) cells was performed as described previously (Woods et al. 2009). Cells were grown to approximately 90% confluency and subcultured in medium containing 0.35 μg/mL puromycin. We measured luciferase activity using the Dual-Luciferase Reporter Assay System (Promega, Madison, WI, USA) according to the manufacturer’s protocol. The luciferase activity was normalized to cell viability, which was determined using a Non-Radioactive Cell-Proliferation Assay Kit (Promega).

### Intracellular peroxide determination

Intracellular peroxide levels were measured by flow cytometry (FACSort; Becton Dickinson, San Jose, CA, USA) using the fluorescent probe 5-(and-6)-chloromethyl-2′, 7′-dichlorodihydrofluorescein diacetate, acetyl ester (Molecular Probes, Eugene, OR, USA) as described previously (Pi et al. 2007).

### Measurement of intracellular glutathione (GSH)

Cells were sonicated in cold phosphate-buffered saline (PBS) immediately after collection, followed by centrifugation at 12,000 × *g* for 5 min. The resulting supernatants were used for measurement of oxidized GSH (GSSG) and total GSH. Samples for GSSG measurement were immediately mixed with thiol-scavenging reagent M2VP (1-methyl-2-vinyl-pyridium trifluoromethane sulfonate) after separation. Levels of total GSH (intracellular GSH + GSSG) and GSSG in cells were measured immediately after collection using the BIOXYTECH GSH/GSSG-412 kit (OxisResearch, Portland, OR, USA) according to the manufacturer’s protocols. The concentrations of intracellular GSH were calculated by the equation


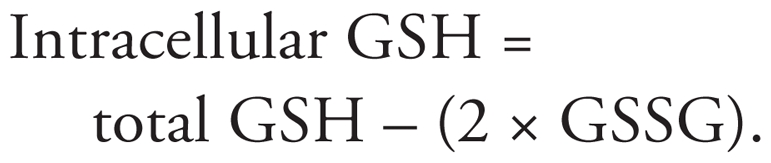


### Measurement of H_2_O_2_-scavenging activity

Cells were washed three times with ice-cold PBS and lysed in the same with 0.5% Protease Inhibitor Cocktail (Sigma) by sonication, followed by centrifugation at 12,000 × *g* for 5 min. The resulting supernatants were used immediately for measurement of H_2_O_2_-scavenging activity as described previously (Pi et al. 2009). Briefly, up to 100 μmol/L of H_2_O_2_ in PBS were incubated with the cell lysates (2 μg protein/μL) for 30 min. The H_2_O_2_ remaining in the solutions was measured using the Amplex Red Hydrogen Peroxide Assay Kit (Invitrogen). The difference in H_2_O_2_ concentrations between lysate-treated and PBS control represents the H_2_O_2_-scavenging activity contributed by cells. Protein concentrations were determined by the Bio-Rad protein assay (Bio-Rad, Hercules, CA, USA) using BSA as a standard.

### Quantitative real-time reverse-transcriptase polymerase chain reaction (RT-PCR) analysis

Total RNA was isolated with TRIzol (Invitrogen), then subjected to cleanup using RNase-Free DNase Set and the RNeasy Mini kit (Qiagen). Quantitative real-time RT-PCR was performed as described previously (Woods et al. 2009). The primers (sequences are shown in Supplemental Material, Table S1 (doi:10.1289/ehp.0901608) were designed using Primer Express 3 (Applied Biosystems, Carlsbad, CA, USA) and synthesized by MWG-BIOTECH Inc. (High Point, NC, USA). Real-time fluorescence detection was carried out using an ABI PRISM 7900 Sequence Detector (Applied Biosystems).

### Western blot analysis

Isolation of cell fractions and Western blotting was performed as detailed previously (Pi et al. 2003b; Woods et al. 2009). We obtained antibodies for Nrf2 (sc-13032; 1:500), glucokinase (GCK; sc-7908; 1:1000), glucose transporter 2 (Glut2, sc-9117; 1:1000), potassium inwardly rectifying channel, subfamily J, member 11 (KCNJ11, also termed KIR6.2; sc-11226; 1:500), sulfonylurea receptor 1 (SUR1; sc-25683; 1:1000), and p68 Src-associated protein in mitosis (SAM68; sc-333; 1:500) from Santa Cruz Biotechnology Inc. (Santa Cruz, CA, USA). Antibodies for lamin A (L1293; 1:2500) and β-actin (A1978; 1:2000) were purchased from Sigma.

### Measurements of mitochondrial mass

We determined mitochondrial mass by flow cytometry (FACSort) and confocal microscope using the fluorescent probe MitoTracker green (Molecular Probes). The final concentration of the probe used was 75 nM, and the preloading time was 30 min. In the flow cytometry measurements, dead cells and clumps were eliminated based on forward scatter versus side scatter measurement, and untreated cells provided a source of comparison. We obtained the fluorescence images using a laser scanning confocal microscope (LSM 510 Meta) mounted on an Axiovert 100M microscope (Carl Zeiss, Inc., Thornwood, NY, USA), using a 488-nm laser for excitation and an LP 505 filter for emission. For acquisition, we used Zeiss LSM510 software, version 4.2 SP1 for Windows 2000.

### Oxygen consumption rate (OCR)

OCR was measured by the XF24 Extracellular Flux Analyzer (Seahorse Bioscience, Billerica, MA, USA) as described previously (Wu et al. 2007).

### Measurement of ATP

Cells were washed three times with ice-cold Kreb’s buffer with the same concentrations of glucose as treatments and lysed in ATP-releasing buffer (Sigma), followed by centrifugation at 12,000 × *g* for 5 min. The resulting supernatants were used immediately for measurement of ATP using an ATP Bioluminescent Assay Kit (Sigma).

### Statistical analyses

All statistical analyses were performed using Graphpad Prism 4 (GraphPad Software, San Diego, CA, USA), with *p* < 0.05 taken as significant. More specific indices of statistical significance are indicated in individual figure legends. Data are expressed as mean ± SE. For comparisons among groups, we performed one-way analysis of variance with Bonferroni post hoc testing.

## Results

### Prolonged arsenite exposure inhibits GSIS in pancreatic β cells

**Figure 1:**
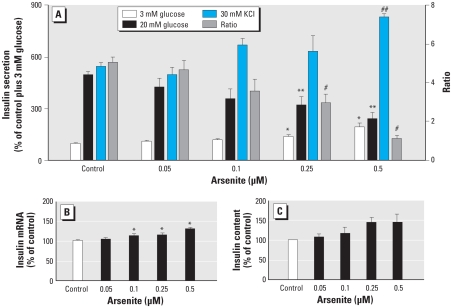
Effect of prolonged arsenite exposure on GSIS in INS-1(832/13) cells. (*A*) Insulin secretion triggered by glucose or KCl measured immediately after a 96-hr arsenite exposure. The level of secreted insulin was normalized by DNA content and expressed as a percentage of control (no arsenite) plus 3 mM glucose; insulin secreted at the 3-mM glucose condition was 0.25 ± 0.06 μg/mg DNA/30 min. The ratio represents insulin secreted at a dose of 20 mM glucose divided by that at 3 mM glucose. Values shown are mean ± SE of two to five independent experiments. (*B*) Gene expression of insulin (cycle threshold value of control = 14). (*C*) Insulin content in whole-cell lysates normalized by DNA content and expressed as the percentage of control (54.0 ± 3.4 μg/mg DNA). *p < 0.05 vs. control plus 3 mM glucose. **p < 0.05 vs. control plus 20 mM glucose. ^#^*p* < 0.05 vs. ratio of control. ^##^*p* < 0.05 vs. control plus KCl.

INS-1(832/13) cells are highly responsive to increasing glucose concentrations and have been used widely to evaluate mechanisms of GSIS *in vitro* (Newgard et al. 2002; Pi et al. 2007). As shown in Figure 1A, exposure of INS-1(832/13) cells to arsenite at noncytotoxic concentrations [see Supplemental Material, Figure 1 (doi:10.1289/ehp.0901608)] for 96 hr resulted in a dose-dependent reduction in insulin secretion in response to glucose stimulation, whereas potassium chloride (KCl), which leads directly to cell membrane depolarization and opening of voltage-operated Ca^2+^ channels, caused a significant increase of insulin release (Figure 1A) in the cells treated with 0.5 μM arsenite. These results are quite intriguing in that they are highly consistent with our previous data showing a markedly blunted insulin secretion from these cells and from mouse islets after a shorter time of exposure (6–24 hr) to arsenite (Pi et al. 2007) [see also Supplemental Material, Figure 2 (doi:10.1289/ehp.0901608)]. In contrast to the decreased GSIS, basal insulin release was elevated (in the presence of 3 mM glucose) in the arsenite-treated cells (Figure 1A). This result is likely due, at least in part, to the increase in gene expression (Figure 1B) and protein levels (Figure 1C) of insulin in the cells. It should be noted that INS-1(832/13) cells have been stably transfected with gene encoding for human proinsulin and selected for the most robust response to glucose (Hohmeier et al. 2000).

### Arsenite activates the Nrf2-mediated antioxidant response

**Figure 2:**
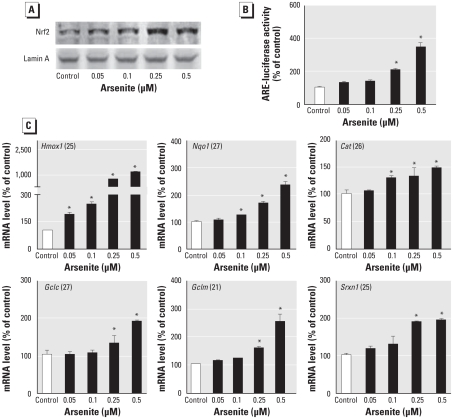
Nrf2-mediated antioxidant response in INS-1(832/13) cells exposed to arsenite for 96 hr. (*A*) Western blot of Nrf2 in isolated nuclear fractions, with lamin A as a loading control. (*B*) Activity of ARE-luciferase reporter. (*C*) Expression of Nrf2-target genes measured by qRT-PCR. The cycle threshold value of that gene in control cells is shown in parentheses after the gene name; *n* = 2–5 independent experiments. *p < 0.05 vs. control.

To determine whether prolonged arsenite exposure activates the Nrf2-mediated antioxidant response, we measured nuclear accumulation of Nrf2 protein, activity of ARE-luciferase reporter, and induction of Nrf2 target genes in the arsenite-exposed cells. As shown in Figure 2A, 96-hr arsenite exposure markedly increased the protein levels of Nrf2 in nuclear fractions in a dose-dependent fashion. The Cignal Lenti ARE reporter is designed to monitor the activity of the antixidant response signal transduction pathway in cultured cells. INS-1(832/13) cells stably transduced with the Cignal Lenti ARE reporter showed dose-dependent induction of luciferase activity after sulforaphane and *tert*-butylhydroquinone treatment [see Supplemental Material, Figure 3 (doi:10.1289/ehp.0901608)], confirming that the cells are responsive to Nrf2 activation. We next examined the ARE activity induced by arsenite. After the ARE reporter cells were exposed to arsenite for 96hr, we observed a dose-dependent increase in ARE-luciferase activity (Figure 2B). Consistent with the ARE-reporter assay, expression of many Nrf2-target genes such as heme oxygenase 1 (*Hmox1*), NAD(P)H: quinone oxidoreductase 1 (*Nqo1*), catalase (*Cat*), γ-glutamate cysteine ligase catalytic subunit (*Gclc*) and regulatory subunit (*Gclm*), and sulfiredoxin 1 (*Srxn1*) were significantly induced (Figure 2C), whereas some genes, including adenine nucleotide translocator (*Ant*), were unaffected (see Supplemental Material, Figure 4). The accumulation of Nrf2 in nuclear fractions, activation of ARE reporter, and significant induction of Nrf2-target genes indicate an activation of Nrf2-mediated adaptive response in the arsenite- exposed cells.

### Arsenite exposure decreases glucose-stimulated peroxide accumulation

**Figure 3:**
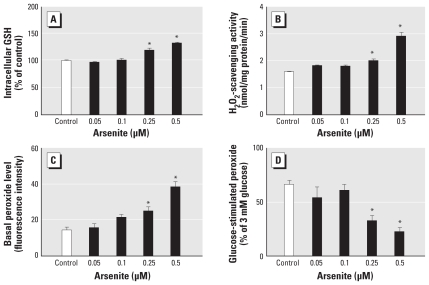
Effect of prolonged arsenite exposure on H_2_O_2_-scavenging activity and glucose-stimulated peroxide production in INS-1(832/13) cells. (*A*) Arsenite increases intracellular GSH levels; GSH levels in whole cell lysates were normalized to protein content and expressed as a percentage of control (54.0 ± 1.7 μmol/g protein). (*B*) H_2_O_2_-scavenging activity in INS-1(832/13) cells. (*C*) Intracellular peroxide level under basal (3 mM glucose) conditions, determined using flow cytometry with fluorescence probe CM-H_2_DCFDA. (*D*) Glucose-stimulated net percentage increase in peroxide level. For each arsenite concentration, the net percentage increase was determined by the following expression: [(peroxide fluorescence at 20 mM glucose – peroxide fluorescence at 3 mM glucose) ÷ peroxide fluorescence at 3 mM glucose × 100]. *p < 0.05 compared with control.

GSH is the most important, as well as most abundant, redox buffer in cells (Jones 2002). In its reaction to scavenge peroxides, GSH is converted by oxidation to GSSG. This disulfide can be reduced back to GSH by glutathione reductase. In keeping with the activation of Nrf2-mediated antioxidant response, evidenced by enhanced expression of Nrf2-target genes (Figure 2C), intracellular GSH (Figure 3A) and intracellular H_2_O_2_-scavenging activity (Figure 3B) were dose dependently increased by arsenite exposure. However, we found no enhanced GSSG level in the arsenite-treated cells (data not shown). In the presence of 3 mM glucose, the basal intracellular peroxide level was significantly increased by arsenite exposure (Figure 3C), which may have contributed to the increased basal insulin secretion (Figure 1A). In contrast to the enhanced basal intracellular peroxide level, the net percentage increase in glucose-stimulated intracellular peroxide production was markedly inhibited in arsenite-exposed cells (Figure 3D), which are positively correlated with their decreased GSIS (Figure 1A). These findings suggest that arsenite-induced oxidative stress triggers cellular adaptive responses, including Nrf2 activation and subsequent enhancement in cellular H_2_O_2_-scavenging activity, which might dampen glucose-triggered ROS signaling that mediates GSIS. Consistent with this notion, INS-1(832/13) cells challenged with another Nrf2 activator, sulforaphane, or overexpressed Nrf2 exhibit a modest but significant decrease in GSIS [see Supplemental Material, Figure 5 (doi:10.1289/ehp.0901608)].

### Effects of arsenite exposure on the consensus GSIS pathway

**Figure 4:**
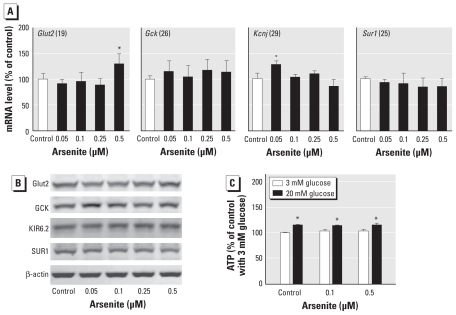
Effect of prolonged arsenite exposure on the classic GSIS pathway. Expression of genes (*A*) and proteins (*B*) in INS-1(832/13) cells exposed to arsenite for 96 hr, measured by qRT-PCR and Western blotting, respectively. The cycle threshold value of that gene in control cells is shown in parentheses after the gene name. (*C*) ATP levels under low and high glucose conditions; values are expressed as a percentage of control cells with low (3 mM) glucose, which was determined as 310.9 ± 9.8 nmol/mg DNA. Data in *A–C* represent three independent experiments. *p < 0.05 compared with control (*A*) or 3 mM glucose in the same cells (*C*).

**Figure 5:**
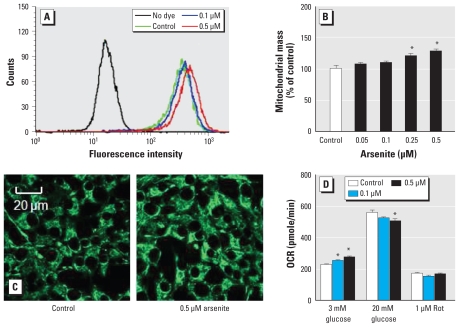
Effect of prolonged arsenite exposure (0.1 μM or 0.5 μM) on mitochondrial mass and OCR in INS-1(832/13) cells exposed to arsenite for 96 hr. (*A*) Measurements of mitochondrial mass using MitoTracker green with flow cytometry. (*B*) Quantitative results of (*A*). Mitochondrial mass is expressed as a percentage of control cells; values represent three to five independent experiments. (*C*) Representative confocal images of mitochondrial mass in cells stained with MitoTracker green. (*D*) OCR under low- and high-glucose conditions. Rotenone (Rot, inhibitor of complex I) was used as a negative control. *p < 0.05 vs. control.

The relationship between glucose stimulation and insulin secretion from the pancreatic β cells is linked with glucose uptake and metabolism as well as with ATP production. In contrast to the dramatic decrease of GSIS in arsenite-exposed INS-1(832/13) cells, no decreased gene and protein expression of major glucose transporter Glut2 and metabolism enzyme Gck was observed (Figure 4A,B). In the consensus model of GSIS, the ATP-sensitive potassium (K_ATP_) channel is an important component in the pathway. The K_ATP_ channel is composed of four units of inwardly rectifying K channel subunit KIR6.2, which is encoded by the *Kcnj11* gene, and four copies of regulatory subunit SUR1 in an octameric complex. SUR1 is a member of the ATP-binding cassette super family. SUR receptor confers the sensitivity of KIR6.2 to ATP/ADP and to pharmacological agents, such as sulfonylurea and diazoxide, that close or open the K_ATP_ channels (Henquin 2000; Wollheim and Maechler 2002). In the present study, the gene and protein expressions of SUR1 and KIR6.2 showed no significant decrease in the arsenite-exposed cells (Figure 4A,B), although the activity of the K_ATP_ channel was not directly determined. ATP production is the primary regulator of K_ATP_; thus, the ATP levels under low- and high-glucose conditions were determined in the arsenite-exposed cells. However, no decrease in glucose-stimulated ATP production was observed in the cells (Figure 4C). Mitochondria have been recognized as some of the most important organelles that regulate GSIS in pancreatic β cells. In contrast to the significant reduction in GSIS, arsenite exposure did not decrease, but dose dependently enhanced, mitochondrial mass (Figure 5A–C). Consistent with this finding, OCR under basal condition (in the presence of 3 mM glucose) was dose dependently increased by arsenite exposure (Figure 5D), although 20 mM glucose-stimulated OCR seemed to show a dose-dependent decrease. The results of these experiments suggest that the impaired GSIS of INS-1(832/13) cells caused by prolonged arsenite exposure is not associated with the consensus GSIS pathway.

## Discussion

**Figure 6:**
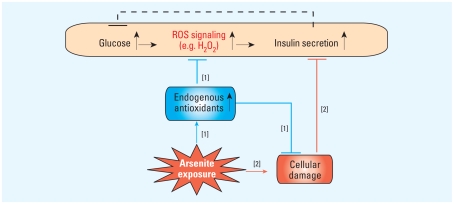
Potential mechanisms involved in decreased GSIS caused by arsenic exposure in pancreatic β cells. [1] Low level of arsenic exposure activates the antioxidant response and thus protects cells from oxidative damage; however, the enhanced antioxidant activities could negatively affect ROS signaling that may contribute to GSIS. [2] A high level of arsenic might result in cellular damage and directly diminish β-cell function.

The development of T2D is usually associated with a combination of β-cell dysfunction and insulin resistance. Normal healthy β cells can compensate for insulin resistance by increasing insulin secretion or β-cell mass. Insufficient compensation ultimately leads to the onset of glucose intolerance and T2D (Kajimoto and Kaneto 2004). Although the precise molecular mechanisms of β-cell dysfunction in T2D are not completely understood, oxidative stress has been increasingly implicated in the pathogenesis of progressive β-cell failure (Kajimoto and Kaneto 2004; Robertson and Harmon 2006). Our previous studies provided evidence that H_2_O_2_ derived from glucose metabolism is one of the metabolic signals for insulin secretion (Pi et al. 2007). In contrast, endogenous antioxidant enzymes that can be induced by various oxidative stress conditions may function as negative regulators for ROS signaling and thus GSIS (Pi et al. 2010; Zhang et al. 2010). In this study, prolonged exposure of INS-1(832/13) cells to low levels of arsenite led to decreased GSIS in a dose-dependent manner. Consistent with this hypothesis, intracellular GSH and H_2_O_2_-scavenging activity were significantly increased in the arsenite-treated cells. The increased antioxidant activity significantly inhibited net glucose-stimulated intracellular peroxide production, which is involved in GSIS. These studies suggest that low levels of arsenic trigger a cellular adaptive response, which impairs ROS signaling involved in GSIS, and thus disturbs β-cell function (Figure 6).

Cellular metabolism is the process of substrate uptake (oxygen, glucose, fatty acids) and energy conversion through a series of enzymatically controlled oxidation/reduction reactions. These reactions are executed through a series of intracellular biochemical processes (glycolysis, Krebs cycle, electron transport, and oxidative phosphorylation) resulting in the production of ATP and the release of heat and chemical by-products (lactate and CO_2_) into the extracellular environment (Wu et al. 2007). GSIS is regulated by the rate of glucose metabolism within β-cells. After its initial uptake and phosphorylation, glucose metabolism involves both cytosolic and mitochondrial processes and generates signals leading to insulin secretion (Newgard and McGarry 1995). It has become established in the field that glycolytic and oxidative processes leading to an increased ATP/ADP ratio are key transduction events in β-cell signaling. However, the precise signals that couple glucose catabolism to insulin secretion are still incompletely understood. The consensus model explaining how glucose generates a triggering signal in β cells involves the following sequence of events: entry of glucose by facilitated diffusion into the cell, metabolism by oxidative glycolysis, rise in ATP/ADP ratio, closure of K_ATP_ channels, depolarization of the plasma membrane potential, opening of voltage-operated Ca^2+^ channels, influx of Ca^2+^, rise in cytosolic Ca^2+^, and activation of exocytotic machinery for release of insulin (Henquin 2000). Improper function of any of these components can impair GSIS. However, in the present study, we found no clear evidence that the consensus GSIS pathway, including expression of Glut2, Gck, and K_ATP_, and glucose-stimulated ATP production, is affected by arsenite exposure, suggesting that a different mechanism is involved in the impairment. Mitochondria are important organelles that regulate GSIS in pancreatic β cells. However, arsenite exposure did not decrease—but dose dependently enhanced—mitochondrial mass. This finding further suggests that the decreased GSIS caused by prolonged arsenite exposure is unlikely related to the impairment of the consensus GSIS pathway. It should be noted that arsenate may uncouple mitochondrial oxidative phosphorylation and decrease ATP generation and thus has the potential to directly impair GSIS. However, the concentration required is much higher than environmentally relevant levels (Tseng 2004). Importantly, in the current study no reduction in ATP production was observed in the arsenite-treated cells.

ROS clearly possess the capacity to behave in a random and destructive fashion and can directly or indirectly disturb physiological functions of many cellular macromolecules such as DNA, protein, and lipids, and activate cellular stress-sensitive signaling pathways. Having evolved in an oxygen environment, most cells have acquired intricate defense mechanisms to counteract oxidative stress and defend against ROS toxicity. Among them, induction of a family of antioxidant/detoxification enzymes that enhance cellular ROS-scavenging capacity is a key element in the maintenance of cellular redox homeostasis and in reducing oxidative damage (Kobayashi and Yamamoto 2006). Accumulating data indicate that these antioxidant genes are coordinately regulated through consensus elements called AREs in their 5′-flanking promoter regions (Nguyen et al. 2003). Nrf2, a member of the bZIP family of transcription factors, is a central regulator of both constitutive and inducible ARE-related gene expression (Itoh et al. 2004). Supporting its importance in antioxidant response is the finding that *Nrf2*-knockout mice show a deficiency in this coordinated gene regulatory program and have a higher susceptibility to oxidative damage and chemical carcinogenesis. Thus, the Nrf2-mediated antioxidant response represents a critically important cellular defense mechanism that serves to maintain intracellular redox homeostasis and limit oxidative damage (Itoh et al. 2004; Nguyen et al. 2003). In spite of this protective role, the Nrf2-mediated antioxidant response has the potential to bring an undesirable effect. In the case where ROS serves as a signal, it could dampen such a signal triggered, for instance, by glucose oxidation. Therefore, we propose that Nrf2-mediated antioxidant response plays a paradoxical role in insulin secretion (Pi et al. 2010). On one hand, it protects β cells from oxidative damage and possible cell death, thus minimizing oxidative damage-related impairment of insulin secretion. On the other hand, because ROS signaling triggered by glucose could be an important component of the machinery of insulin secretion, the induction of endogenous antioxidants in the presence of oxidative stress may blunt this signal, resulting in reduced GSIS (Figure 6). We envisage the following scenarios for arsenic- induced impairment of β-cell function. Under low-level oxidative stress, such as low-dose arsenite exposure, β cells can adapt to the condition adequately by activating the Nrf2-ARE system, thereby keeping oxidative damage/cell death-related impairment of GSIS at a minimum. However, on a chronic basis, an increase in the level of endogenous antioxidants may interfere with glucose-dependent ROS signals that we postulate directly contribute to GSIS. Under excessive oxidative stress or high dose of arsenic exposure, the Nrf2 system may be overwhelmed and unable to adequately compensate, resulting in irreversible oxidative damage. In this situation, excessive oxidative damage and possible cell death become the primary cause for the impaired GSIS; thus, the machinery of GSIS in β cells is likely to be critically sensitive to all levels of arsenic. These considerations are compatible with the view that oxidative stress may contribute to both early and late phases of β-cell failure in T2D.

Taken together, our data indicate that low concentrations of arsenite cause oxidative stress and cellular adaptive response, including Nrf2 activation, in β cells, and such a cellular adaptive response to arsenite is associated with impairment of GSIS. Although the etiology of T2D is still unclear to date, therapeutic approaches have focused on medication and lifestyle modification. The role of environmental exposures must also be considered in the future.
